# Diversity and Structural Composition of Bacterial Communities in Mineral Springs of the Andean–Amazonian Transition Zone in Northern Peru

**DOI:** 10.3390/microorganisms14092106

**Published:** 2026-09-20

**Authors:** Damaris Leiva-Tafur, Liz M. Cumpa-Velásquez, Lorenzo Culqui, Carmen N. Vigo, Cristian Hurtado Burga, Deisy Iliquin Chavez, Oscar Andrés Gamarra-Torres, Manuel Oliva-Cruz

**Affiliations:** 1 Escuela de Posgrado, Programa Doctoral en Ciencias para el Desarrollo Sustentable, Facultad de Ingeniería Zootecnista, Biotecnología, Agronegocios y Ciencia de Datos, Universidad Nacional Toribio Rodríguez de Mendoza de Amazonas, Chachapoyas 01001, Peru; oscar.gamarra@untrm.edu.pe; 2 Instituto de Investigación para el Desarrollo Sustentable de Ceja de Selva (INDES-CES), Universidad Nacional Toribio Rodríguez de Mendoza de Amazonas (UNTRM), Chachapoyas 01001, Peru; marjory.cumpa@untrm.edu.pe (L.M.C.-V.); lorenzo.culqui@untrm.edu.pe (L.C.); canavimes7694@gmail.com (C.N.V.); 3 Facultad de Ingeniería Ambiental, Biosistemas y de la Energía, Universidad Nacional Toribio Rodríguez de Mendoza de Amazonas (UNTRM), Chachapoyas 01001, Peru; 7177649122@untrm.edu.pe (C.H.B.); iliquinchavezdeisy079@gmail.com (D.I.C.)

**Keywords:** biogeochemical cycling, extremophiles, functional diversity, hydrogeochemical heterogeneity, microbial bioprospecting

## Abstract

Mineral springs in the Andean–Amazonian transition represent poorly explored aquatic ecosystems whose hydrogeochemical heterogeneity may favor the establishment of specialized microbial communities. In this study, bacterial communities in sediments from 19 mineral springs in northern Peru were characterized using 16S rRNA gene sequencing, and their relationships with hydrogeochemical conditions and predicted functional potential were evaluated. The springs exhibited temperatures ranging from 15.1 to 38.2 °C and total dissolved solids (TDS) concentrations ranging from 0.022 to 162.5 mg L^−1^, reflecting marked variation in thermal and mineralization conditions. Bacterial communities were dominated by Desulfobacteraceae, Desulfosarcinaceae, Chlorobiaceae, Nitrosomonadaceae, Nitrospiraceae, and Rhodobacteraceae, including representative genera such as *Halanaerobium*, *Chlorobium*, *Thiothrix*, and *Sulfurovum*. Although alpha diversity did not differ significantly among hydrogeochemical categories, beta diversity analyses indicated compositional differentiation among thermal categories, while RDA revealed a significant multivariate association between bacterial community composition and the combined hydrogeochemical gradient (F = 1.0934, *p* = 0.035). Co-occurrence network analysis identified *Desulfotignum*, *Roseimarinus*, *Halanaerobium*, and *Anaerophaga* among the most highly connected taxa. PICRUSt2 predicted functional potential associated with carbon fixation, nitrogen and sulfur transformations, phosphorus acquisition and polyphosphate-related metabolism, and ferrous iron uptake. These findings provide the first integrated characterization of these bacterial communities and establish a baseline for future research on microbial ecology, biogeochemical cycling, and environmental biotechnology in Andean–Amazonian ecosystems of Peru.

## 1. Introduction

Microbial communities play an essential role in natural aquatic ecosystems by actively participating in biogeochemical cycles, mineral transformation, and the regulation of water quality [1]. In particular, mineralized waters and geothermal systems are unique environments in which physicochemical factors such as temperature, pH, electrical conductivity, and ionic composition serve as key environmental filters shaping the structure and taxonomic diversity of microbial communities [2,3]. Because of these characteristics, these systems have been widely recognized as natural laboratories for studying microbial ecology and adaptation to extreme conditions [4,5].

The development of molecular tools, particularly 16S rRNA gene sequencing and metagenomic approaches, has led to significant advances in the taxonomic characterization of microbial communities inhabiting mineralized waters and natural springs [6,7,8]. Studies conducted in geothermal systems across different regions of the world have demonstrated that microbial diversity exhibits consistent patterns of bacterial and archaeal dominance that are closely associated with specific physicochemical gradients. These investigations have established a solid conceptual framework linking microbial community structure to the prevailing environmental conditions in mineralized aquatic systems [9,10].

In mountainous and tropical regions such as the Andes, the interaction of active tectonics, complex geology, and pronounced altitudinal gradients generates marked hydrogeochemical heterogeneity in natural mineralized waters [11]. Studies conducted in Andean geothermal ecosystems have shown that these environments harbor highly specialized microbial communities adapted to high concentrations of dissolved salts and trace metals, as well as broad thermal gradients, reflecting strong environmental selection driven by extreme hydrogeochemical conditions [12,13,14]. However, most of these investigations have focused on high-altitude saline lakes or well-characterized hot springs, leaving other types of natural mineralized waters in transitional zones between the Andes and the Amazon relatively unexplored [15,16,17].

In Peru, research on mineralized waters and natural springs has historically focused on their physicochemical and sanitary characterization [18,19]. Although recent studies have begun to incorporate 16S rRNA-based taxonomic approaches in some geothermal systems across the country, the available information remains fragmented and geographically limited [20,21]. In particular, the Andean–Amazonian region of northern Peru remains underrepresented in studies of the microbial ecology of natural mineralized waters, despite its high environmental heterogeneity, pronounced altitudinal gradients, and complex geology resulting from the interaction between the Andes Mountain Range and the Amazon Basin, factors recognized as key drivers of aquatic microbial community structure [22]. Unlike intensively studied geothermal regions worldwide, where comprehensive investigations have enabled evaluation of large-scale ecological patterns [23], the information available for Peru is based primarily on local, geographically restricted assessments, thereby limiting understanding of microbial biogeographic patterns in these ecosystems [24]. Beyond addressing this fundamental ecological knowledge gap, characterizing microbial diversity in mineral springs may also have practical relevance. Microorganisms adapted to thermal and mineral-rich conditions may represent potential sources of enzymes and metabolic capabilities of interest for environmental and biotechnological applications [25,26,27]. Furthermore, establishing baseline information on microbial diversity and its relationships with hydrogeochemical conditions can support future ecological assessment and conservation of these poorly characterized aquatic ecosystems. Despite their ecological and potential practical relevance, significant knowledge gaps remain regarding the microbial communities inhabiting mineral springs of the Andean–Amazonian region. Therefore, the present study aimed to explore the bacterial diversity associated with mineral springs located in the Andean–Amazonian transition of northern Peru. To achieve this, the physicochemical characteristics and trace element concentrations of these ecosystems were first characterized. Subsequently, the composition and diversity of bacterial communities at the family and genus levels were evaluated using 16S rRNA gene sequencing. Finally, the relationships between the physicochemical characteristics of the mineral springs and patterns of bacterial diversity and community structure were analyzed to identify the primary environmental factors driving microbial community distribution.

## 2. Materials and Methods

### 2.1. Study Area

The study area encompassed 19 mineral springs distributed across six of the seven provinces of the Amazonas region—Bagua, Bongará, Chachapoyas, Luya, Rodríguez de Mendoza, and Utcubamba—within the Andean–Amazonian transition zone of northern Peru, where the interaction among mountainous terrain, tectonic activity, and high precipitation creates a complex hydrogeological system [28,29]. In this region, compressional deformation associated with the subduction of the Nazca Plate has generated deep faults and fractures that serve as preferential pathways for the circulation of long-residence-time groundwater [30]. The topography controls the regional hydrological dynamics: fractured highlands function as recharge areas, whereas valley bottoms, lithological contacts, and structural escarpments act as discharge zones where mineral springs emerge [31]. This setting is further influenced by pronounced climatic heterogeneity, ranging from relatively dry to superhumid conditions, which contributes to the spatial hydrogeochemical variability observed throughout the region [32,33].

**Figure 1:**
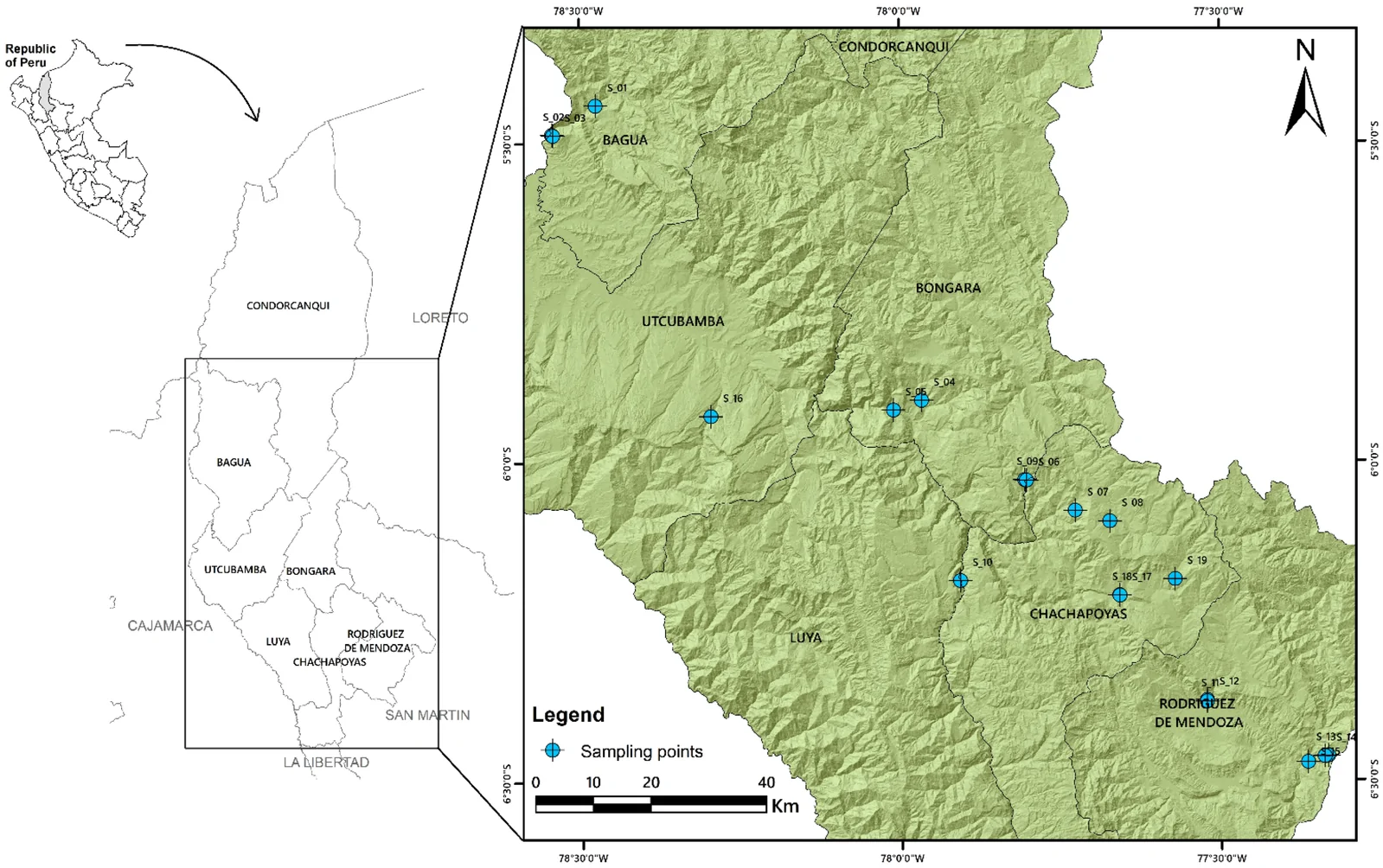
Spatial Distribution of Mineral Spring Sampling Sites in the Amazonas Region, Peru. Some sampling points overlap because of their close geographic proximity at the scale of the map.

Mineral spring exploration was carried out using an exploratory survey approach in the Amazonas region of northern Peru. The survey initially considered eight mineral springs previously documented by the Geological, Mining and Metallurgical Institute of Peru (INGEMMET) [34]. Based on information obtained from local communities, eleven additional mineral springs were identified and subsequently confirmed through direct field inspections of each spring outcrop. Consequently, a total of nineteen mineral springs were identified and evaluated in this study. Although Condorcanqui Province is displayed in Figure 1 as part of the administrative division of the Amazonas region, no mineral springs from this province were included in the study. The geographic coordinates and elevation of each of the 19 sampling sites are provided in Supplementary Table S1. Coordinates are reported using the WGS84/UTM coordinate reference system (Zones 17S and 18S).

### 2.2. Sample Collection and Determination of Physicochemical Parameters and Trace Elements

In situ physicochemical parameters, including pH, temperature, dissolved oxygen (DO), and electrical conductivity (EC), were measured directly at each mineral spring using a WTW Multi 3620 IDS multiparameter instrument (Xylem Analytics Germany Sales GmbH & Co., KG, Weilheim, Germany), with measurement accuracies of ±0.004 pH units, ±0.5% for EC and DO, and ±0.2 °C for temperature. Total dissolved solids (TDS), alkalinity, chloride, hardness, sulfate, and trace elements (As, Ca, Cu, Fe, K, Mg, Mn, Na, Pb, Sb, Sr, and Zn) were quantified following the standardized methods described in the Standard Methods for the Examination of Water and Wastewater [35].

Water samples were collected in pre-conditioned 1 L high-density polyethylene (HDPE) containers following standardized protocols for the sampling and preservation of natural waters (ISO 5667-3, 2024) [36]. For trace element analysis, 100 mL HDPE bottles were used after prior decontamination by immersion in a 10% nitric acid solution (1 M), followed by rinsing with ultrapure water. Trace elements were quantified using inductively coupled plasma mass spectrometry (ICP-MS) in accordance with the U.S. Environmental Protection Agency (EPA) guidelines for the analysis of metals in aqueous matrices [37].

For molecular analyses, three surface sediment subsamples were collected aseptically from the central area of each mineral spring using sterile 50 mL Falcon tubes. The subsamples were subsequently homogenized in the field to obtain a single composite sample representative of each spring, thereby reducing small-scale spatial heterogeneity and providing a more representative characterization of the resident microbial community. One composite sediment sample per mineral spring was therefore used for DNA extraction and sequencing, resulting in 19 independent site-level samples; no within-spring biological replicates were sequenced. The composite samples were preserved under a cold chain at 4 °C and immediately transported to the Molecular Biology Unit of the Plant Health Laboratory for DNA extraction, whereas water samples were transported to the Water and Soil Research Laboratory for physicochemical analyses. Field instruments were calibrated daily using certified standard solutions, and physicochemical analyses were performed in triplicate for each water sample to ensure the accuracy, reproducibility, and traceability of the results, in accordance with quality assurance and quality control guidelines established under ISO/IEC 17025:2017 [38].

### 2.3. Classification of Mineral Springs Based on Their Physicochemical Characteristics

The hydrogeochemical classification of the mineral springs used in this study was based on the framework previously developed for these same aquatic systems in the Amazonas region [39], which integrates physicochemical criteria widely recognized in the literature on mineral waters and geothermal systems. The waters were classified according to pH as acidic (pH < 7) or alkaline (pH ≥ 7), and according to temperature as cold (<20 °C), hypothermal (20–30 °C), and mesothermal (30–40 °C), following approaches commonly applied in hydrogeochemical studies of geothermal environments [40,41].

The degree of mineralization was determined based on total dissolved solids (TDS) concentration, classifying the waters as oligomineral (<100 mg/L), low mineralization (100–250 mg/L), moderate mineralization (250–500 mg/L), high mineralization (500–1000 mg/L), or very high mineralization (>1000 mg/L), according to the criteria proposed by Lourenço et al. [42]. These mineralization classes, rather than individual trace metal concentrations, were used as physicochemical categories in the subsequent microbial diversity analyses. Likewise, water hardness, expressed as CaCO_3_, was categorized as very soft (<100 mg/L), soft (100–200 mg/L), hard (200–300 mg/L), and very hard (>300 mg/L), following the criteria described by Sawyer et al. [43] and the World Health Organization [44].

### 2.4. Genomic DNA Extraction and Quality Assessment

Approximately 0.25 g of previously homogenized sediment was used for genomic DNA extraction using the DNeasy^®^ PowerSoil Pro Kit (QIAGEN, Hilden, Germany), following the manufacturer’s instructions. DNA concentration and purity were determined using an Eppendorf BioSpectrophotometer Basic (Eppendorf AG, Hamburg, Germany), while DNA integrity was assessed by 1% agarose gel electrophoresis. Samples that met the established quality criteria for DNA concentration, purity, and integrity were subsequently submitted for high-throughput sequencing, as described in the following section.

### 2.5. 16S rRNA Gene Amplicon Sequencing and Raw Sequence Processing

The V3–V4 hypervariable region of the bacterial 16S rRNA gene was amplified by PCR using the universal primer pair 341F (5′-CCTAYGGGRBGCASCAG-3′) and 806R (5′-GGACTACNNGGGTATCTAAT-3′). Amplicon libraries were prepared and sequenced by Novogene Co., Ltd. (Beijing, China) using an Illumina paired-end 250 bp (PE250) platform (Batch ID: X202SC24060655-Z01-F001).

The raw demultiplexed reads were imported into QIIME 2 (version 2024.6) using the paired-end FASTQ manifest format. All bioinformatic analyses were performed on the High-Performance Computing (HPC) platform at the Universidad Nacional Toribio Rodríguez de Mendoza. Initial sequence quality and read quality distributions were assessed using the demux plugin. Quality filtering, trimming, paired-end read merging, chimera removal, and Amplicon Sequence Variant (ASV) inference were subsequently performed using the DADA2 plugin. To remove low-quality bases from the 3′ ends while maintaining sufficient overlap for paired-end merging, the forward reads were retained at their original length, whereas the reverse reads were truncated to 215 base pairs (bp). Following denoising, representative ASVs were aligned, masked, and used to construct a midpoint-rooted phylogenetic tree through the phylogeny plugin, which incorporates the MAFFT multiple sequence alignment algorithm and the FastTree phylogenetic inference tool. Taxonomic assignment of the resulting ASVs was performed using the feature-classifier plugin with a Naive Bayes classifier pre-trained on the SILVA reference database.

### 2.6. Data Analysis

Redundancy Analysis (RDA) were performed in R (Version 4.6.1) using the vegan package to evaluate the association between bacterial community composition and environmental variation. Prior to constrained ordination, the ASV abundance matrix was Hellinger-transformed. The environmental dataset comprised 18 physicochemical variables, which were standardized to zero mean and unit variance. Because strong multicollinearity was detected among the environmental variables, principal component analysis (PCA) was used as a dimensionality-reduction step. The scores of the first two principal components (PC1 and PC2), which together explained 46.54% of the environmental variance, were subsequently used as constraints in the RDA. The statistical significance of the overall constrained model was assessed using 999 permutations. Original environmental variables were passively fitted onto the RDA ordination using the envfit function to facilitate interpretation of the hydrogeochemical gradients.

Using the microeco package in the R environment, alpha and beta diversity indices were calculated to evaluate the structure of the microbial communities. To assess the influence of environmental factors on microbial community structure, Redundancy Analysis (RDA) was conducted following a statistical refinement procedure. The environmental matrix, comprising 18 variables, was normalized through scaling, and constant variables were removed. Subsequently, Principal Component Analysis (PCA) was applied to reduce data dimensionality and minimize collinearity among the environmental variables. The statistical significance of the model was assessed using a permutation test with 999 permutations.

Co-occurrence networks were constructed at the ASV level using the trans_network class implemented in the microeco package. Before network construction, low-abundance and infrequent ASVs were removed using filter_taxa (rel_abund = 0.001, freq = 0.2), with an additional abundance threshold of 0.001 applied during network inference. After filtering, 21 of the 54,358 ASVs detected in the complete dataset were retained for the correlation analysis. Spearman’s rank correlations were then calculated for all pairwise combinations of these ASVs, resulting in 210 unique comparisons. We used a stringent threshold (ρ > 0.90) to retain only strong positive co-occurrence patterns and to reduce network complexity. To account for multiple comparisons, *p*-values were adjusted using the Benjamini–Hochberg false discovery rate (FDR), and associations were retained when ρ > 0.90 and q < 0.05. Network modules were identified using the Louvain algorithm, while degree and strength were used to describe node connectivity and identify highly connected ASVs as putative hub candidates. The final network was visualized in Gephi Lite, where the node layout was adjusted to facilitate interpretation of the network structure. Because correlation does not necessarily indicate direct ecological interaction, network edges were interpreted as patterns of co-occurrence among ASVs.

Finally, the potential functional composition of the bacterial community was predicted using the PICRUSt2 pipeline (v0.3.8). The complete feature table and representative sequences from the entire dataset were used for functional inference. Output tables containing KEGG Orthology (KO) and Enzyme Commission (EC) annotations were processed to characterize the predicted functional profile. As an overview of the dominant predicted functions, the 20 most abundant KOs were classified according to KEGG Level 2 functional categories and visualized as a heatmap (Supplementary Figure S4). In addition, to specifically examine predicted functions of biogeochemical relevance, 17 KOs associated with carbon fixation, nitrogen and sulfur transformations, phosphorus acquisition and polyphosphate-related metabolism, and ferrous iron uptake were selected from the complete predicted KO abundance table. Functional assignments of the selected KOs were verified against KEGG Orthology. KO abundances were converted to relative abundances within each sample and subsequently standardized across the 19 samples using row-wise Z-scores for heatmap visualization. Samples were arranged sequentially according to the sampling-site identification scheme (S1–S19). Data processing, normalization, and statistical visualizations were performed using the data.table and ggplot2 [45] packages in the R environment [46]. Because PICRUSt2 infers functional profiles from 16S rRNA gene data, these results were interpreted as predicted functional potential rather than direct evidence of gene expression or in situ metabolic activity.

## 3. Results

### 3.1. Physicochemical Characteristics and Hydrogeochemical Classification of Mineral Springs

To characterize the physicochemical properties of the 19 mineral springs evaluated, physicochemical parameters and trace elements were determined (Table 1), revealing marked variability associated with the altitudinal gradient and local hydrogeochemical conditions. The springs were distributed between 384 and 3147 m above sea level, exhibiting differences in water temperature, oxygenation, and mineralization (Supplementary Table S2).

The pH values ranged from 5.20 to 8.72, with a mean of 6.72 ± 0.79, indicating an overall slightly acidic tendency across the mineral springs. Total dissolved solids, hardness, and alkalinity exhibited wide ranges. Among the major ions, sodium and sulfate displayed the highest average concentrations, while calcium and magnesium showed moderate variability. Trace element concentrations were generally low, although some sites exhibited localized increases in aluminum and arsenic. Likewise, microbiological indicators showed heterogeneous distributions among springs, with variable occurrences of total coliforms, fecal coliforms, and *Escherichia coli*.

Part of this physicochemical characterization was previously reported in a study focused on the hydrogeochemical and microbiological quality of mineral springs in the Amazonas region of Peru [39]. In the present study, these data were integrated and analyzed in relation to the bacterial diversity of the mineral springs.

To examine the relationship between mineral water classification and patterns of microbial diversity, the sampled springs were categorized based on their main physicochemical properties. Hydrogeochemical classification was based on temperature, pH, hardness, and degree of mineralization, following criteria widely recognized and accepted in hydrogeochemical and mineral water studies [40].

**Figure 2:**
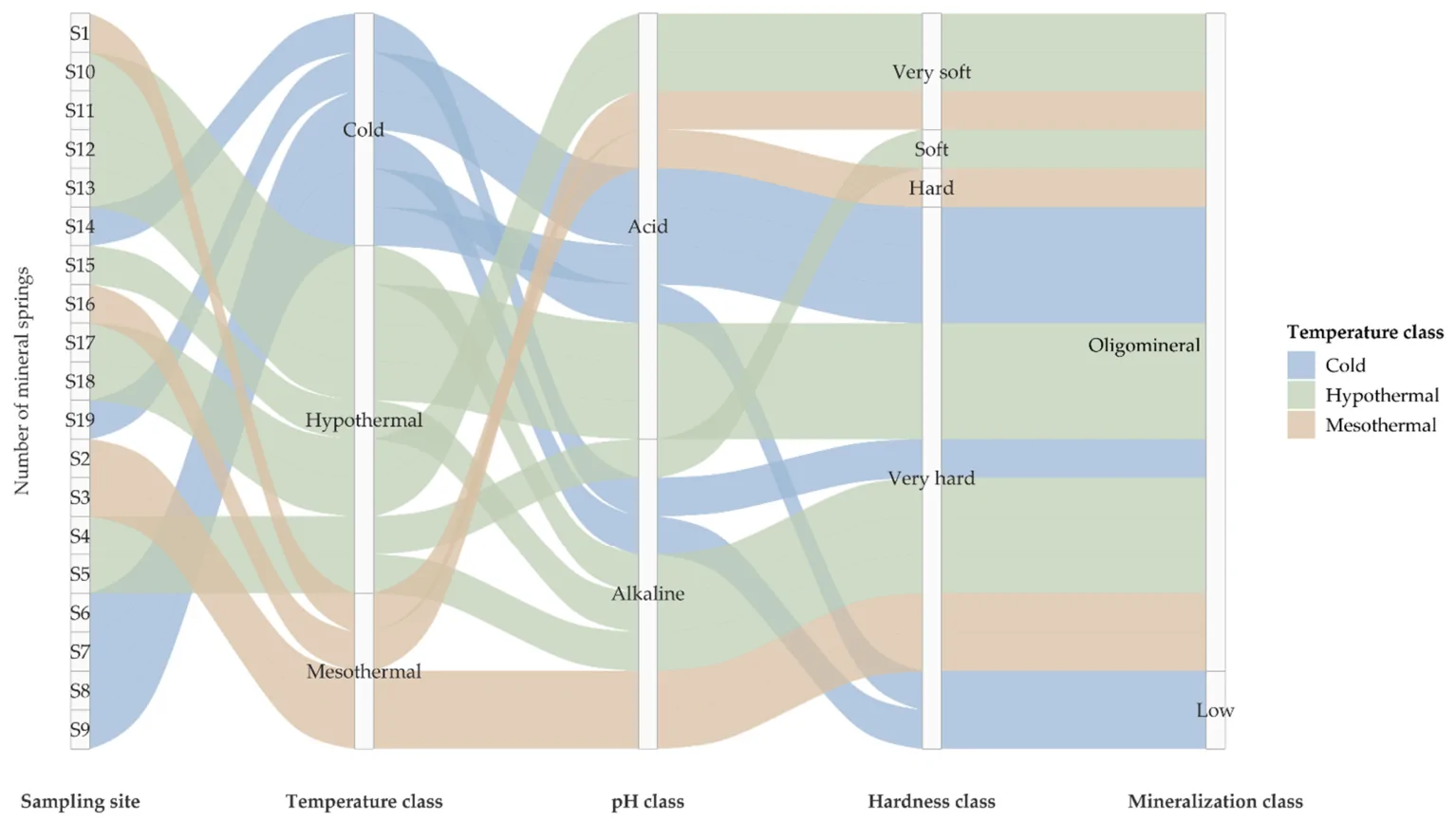
Alluvial diagram showing the classification of mineral springs by temperature, pH, hardness, and mineralization across sampling sites in the Andean–Amazonian transition zone of northern Peru.

The alluvial diagram (Figure 2) illustrates the relationships between the sampling sites and the different physicochemical categories, revealing 11 hydrogeochemical combinations based on temperature, pH, hardness, and degree of mineralization. The most common combination consisted of hypothermal, acidic, very hard, and oligomineral waters, representing the largest proportion of the springs evaluated. Several springs were classified as cold, acidic, very hard, and oligomineral, whereas mesothermal springs were associated with both acidic and alkaline conditions. Based on the established pH classification (acidic, pH < 7; alkaline, pH ≥ 7), 11 of the 19 springs (57.9%) were classified as acidic and 8 (42.1%) as alkaline, demonstrating that acidic conditions were more frequent among the evaluated springs. In terms of hardness, the very hard water category was by far the most prevalent, whereas soft and very soft waters were represented by only a few springs. Oligomineral waters dominated at nearly all sampling sites, with only two springs classified as low-mineralization.

### 3.2. Bacterial Community Diversity and Composition

Sequencing of the 19 mineral spring sediment samples yielded a total of 2,392,838 quality-filtered reads, with per-sample sequencing depth ranging from 79,412 to 173,263 reads (mean = 125,939). A total of 54,358 unique ASVs were detected across the complete dataset. At the individual-sample level, ASV richness ranged from 545 to 6563 ASVs, with a mean of 3179 ASVs per sample. Because individual ASVs could occur in more than one sample, the dataset-wide number represents unique ASVs after pooling all samples, whereas per-sample richness counts each ASV in every sample in which it was detected. Taxonomic assignment based on the SILVA reference database was successful for 89.0% of ASVs at the family level and 79.4% at the genus level.

**Figure 3:**
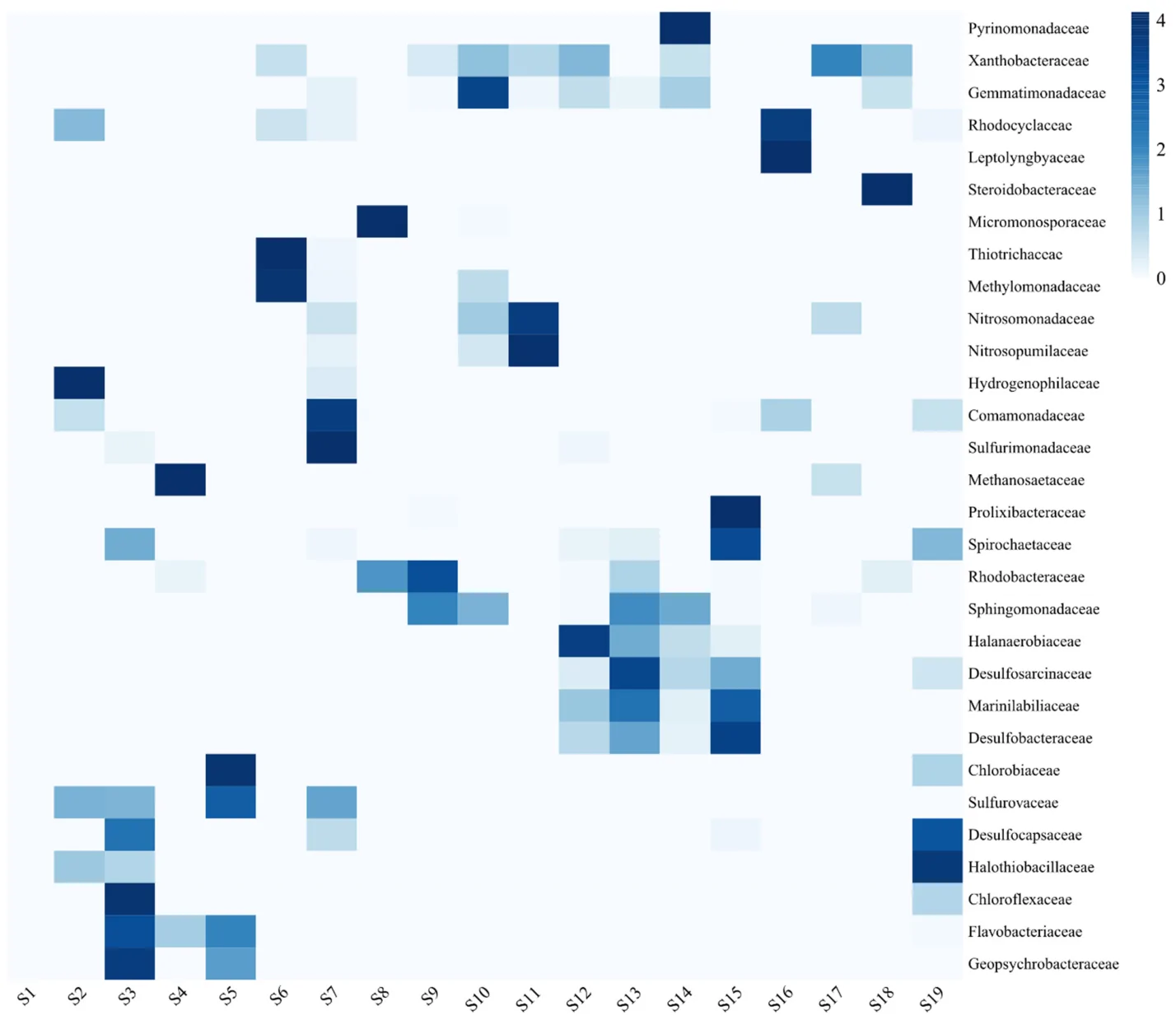
Heatmap of the 30 most abundant bacterial families in mineral springs of the Andean–Amazonian transition zone in northern Peru. Color intensity represents the scaled relative abundance of each bacterial family.

The taxonomic characterization of the bacterial communities was based on the 30 most abundant taxa selected independently at the family and genus levels. At the family level, the top 30 families showed a heterogeneous distribution among the 19 mineral springs, with distinct differences in relative abundance patterns among the sampling sites (Figure 3). The families with the highest relative abundance per site were Hydrogenophilaceae (S2); Chlorobiaceae (S5); Thiotrichaceae (S6); Sulfurimonadaceae (S8); Nitrosomonadaceae and Nitrosopumilaceae (S10–S11); Halanaerobiaceae, Desulfosarcinaceae, Marinilabiliaceae and Desulfobacteraceae (S12–S15); and Desulfocapsaceae, Halothiobacillaceae and Geopsychrobacteraceae (S3 and S19).

At the genus level, the top 30 genera grouped by province showed that the “Others” category (low-abundance taxa) accounted for between 46% (Rodríguez de Mendoza) and 96% (Utcubamba) of the relative abundance (Supplementary Figure S1). Among the named genera, the highest relative abundances corresponded to *Oscillochloris* (~13%), *Sulfuricurvum* (~9%), and *Longispora* (~8%) in Bagua; *Halanaerobium* (~14%) and *Anaerophaga* (~7%) in Bongará; *Halanaerobium* (~8%) in Chachapoyas; *Luteolibacter* (~24%) in Luya; *Chlorobium* (~19%) and *Thiothrix* (~11%) in Rodríguez de Mendoza; and no genus exceeding 5% relative abundance in Utcubamba.

Alpha diversity values varied considerably among the 19 mineral springs (Supplementary Table S1). Evenness ranged from 0.480 to 0.943 (mean = 0.761 ± 0.118), Shannon entropy from 3.851 to 11.122 (mean = 8.009 ± 1.944), the Simpson index from 0.865 to 0.999 (mean = 0.967 ± 0.037), and Faith’s phylogenetic diversity from 47.100 to 229.407 (mean = 140.936 ± 54.026). None of these indices differed significantly among temperature, pH, hardness, or mineralization categories according to Kruskal–Wallis tests (H = 0.000–4.614, *p* = 0.202–1.000; Supplementary Table S3), a pattern also reflected in Supplementary Figure S2.

Beta diversity, by contrast, showed a more defined structure. Pairwise PERMANOVA based on Bray–Curtis dissimilarity detected significant compositional differences between cold and mesothermal springs (pseudo-F = 1.134, q = 0.045) and between hypothermal and mesothermal springs (pseudo-F = 1.096, q = 0.045), while cold and hypothermal springs did not differ significantly from one another (pseudo-F = 0.994, q = 0.421) (Supplementary Table S4; Supplementary Figure S3). No significant differences were found between pH or mineralization categories, nor when community composition was evaluated using Jaccard or UniFrac distances (q > 0.05 for all comparisons).

### 3.3. Environmental Drivers of Bacterial Community Structure (RDA)

Prior to RDA, the standardized environmental dataset was summarized using PCA to reduce dimensionality associated with strong multicollinearity among environmental variables. PC1 and PC2 explained 30.96% and 15.59% of the environmental variance, respectively, accounting for 46.54% cumulatively. PC1 was primarily characterized by chloride (loading = −0.970), electrical conductivity (−0.928), total solids (−0.925), temperature (0.828), and total suspended solids (−0.821), whereas PC2 was mainly characterized by K (0.742), Mg (0.734), pH (0.660), sulfate (0.521), and Al (−0.481). PCA loadings for all 18 environmental variables are provided in Supplementary Table S5.

**Figure 4:**
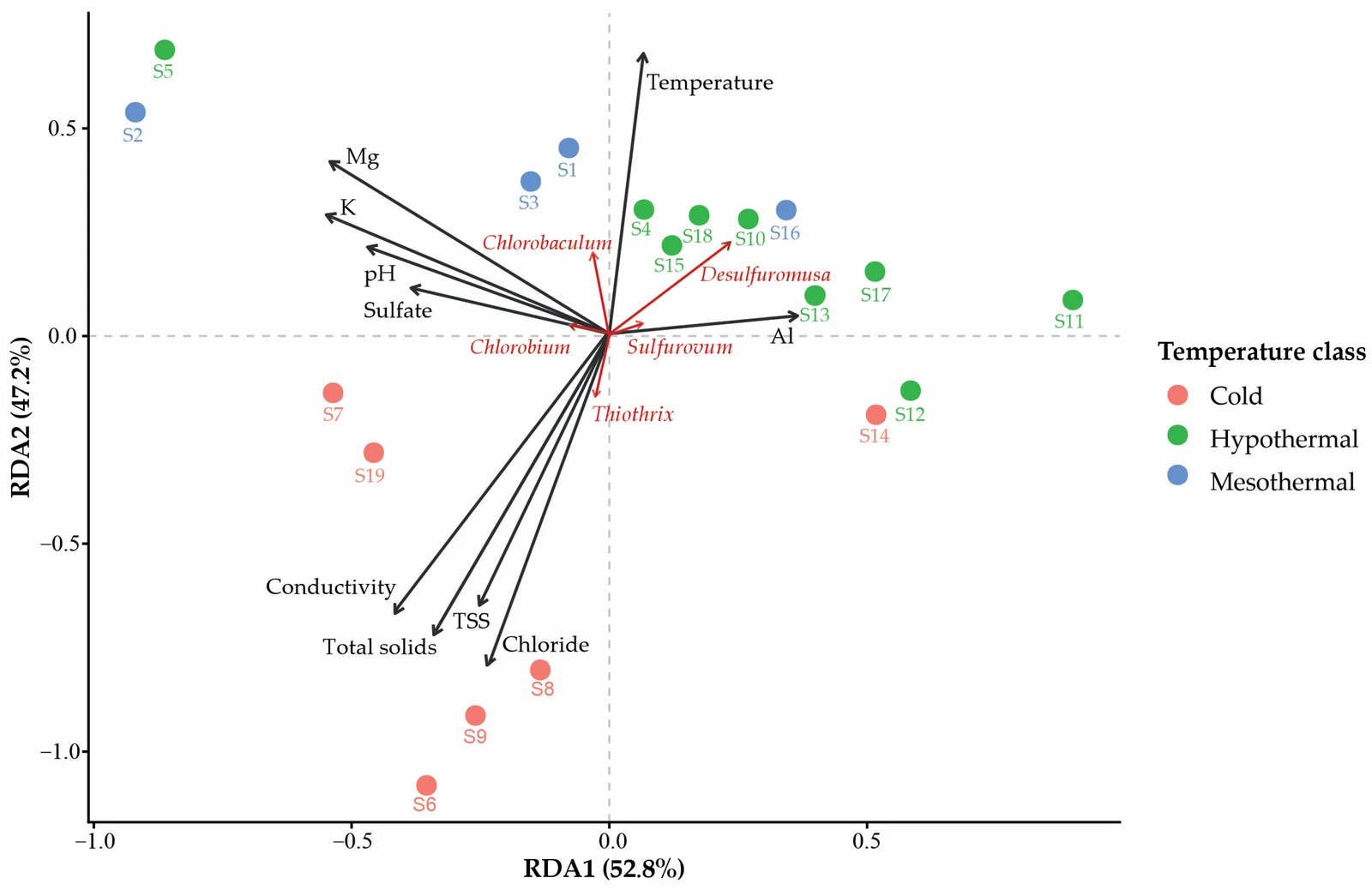
Redundancy analysis (RDA) of Hellinger-transformed bacterial community composition constrained by the first two principal components of the standardized environmental dataset. RDA1 and RDA2 represent 52.8% and 47.2% of the constrained variance, respectively.

The RDA constrained by these two environmental components was statistically significant (F = 1.0934, *p* = 0.035; 999 permutations) and accounted for 12.02% of the total variation in bacterial community composition (R^2^ = 0.120; adjusted R^2^ = 0.010). RDA1 and RDA2 represented 52.8% and 47.2% of the constrained variance, respectively. Original environmental variables were passively fitted onto the ordination to facilitate interpretation of the hydrogeochemical gradients shown in Figure 4.

### 3.4. Microbial Co-Occurrence Network

**Figure 5:**
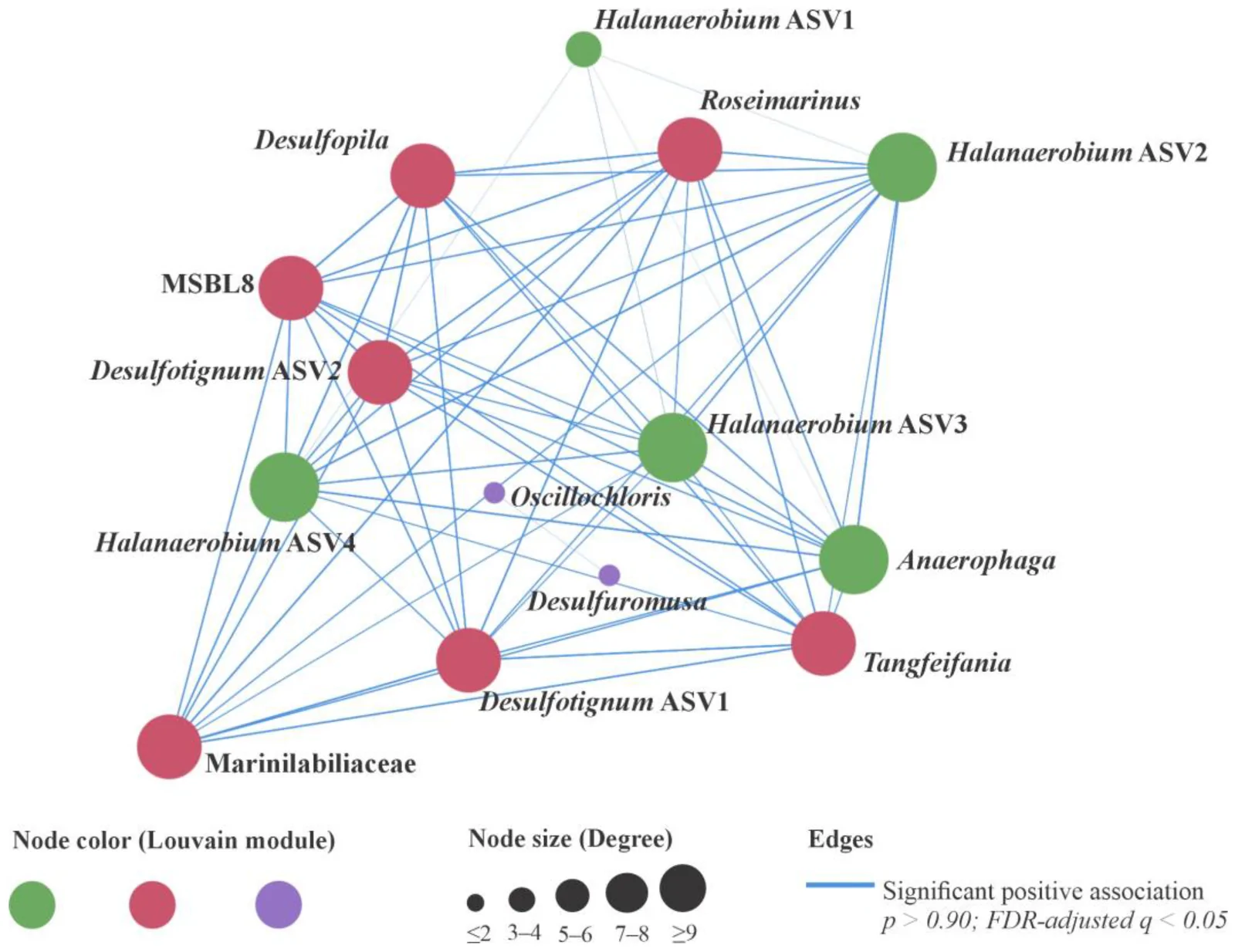
Co-occurrence network of bacterial ASVs in the studied mineral springs. Nodes represent 14 ASVs connected by 60 significant positive Spearman associations (*p* > 0.90; FDR-adjusted q < 0.05).

After abundance and frequency filtering, 21 ASVs were retained for the co-occurrence analysis, resulting in 210 unique pairwise Spearman correlations. Of these, 60 strong positive associations met the criteria of ρ > 0.90 and FDR-adjusted q < 0.05. These associations involved 14 connected ASVs (Figure 5). The network was organized into three modules identified by the Louvain algorithm, containing 2, 5, and 7 ASVs, respectively. Node degrees ranged from 1 to 11, showing differences in connectivity among ASVs. The highest degree values were found for three ASVs assigned to *Halanaerobium* and one ASV assigned to *Anaerophaga* (degree = 11). Several ASVs affiliated with *Desulfopila*, *Desulfotignum*, *Tangfeifania*, MSBL8, *Roseimarinus*, and *Marinilabiliaceae* also showed high connectivity (degree = 10). In contrast, *Desulfuromusa* and *Oscillochloris* had the lowest connectivity (degree = 1) and occupied more peripheral positions in the network. Taxonomic annotation of the 14 connected ASVs identified five bacterial phyla: Halanaerobiaeota, Desulfobacterota, Bacteroidota, Cloacimonadota, and Chloroflexi. Halanaerobiaeota, Desulfobacterota, and Bacteroidota were represented by four ASVs each, whereas Cloacimonadota and Chloroflexi were represented by one ASV each. The highly connected ASVs were mainly affiliated with the first three phyla, indicating their central position within the observed co-occurrence structure.

### 3.5. Functional Prediction of the Bacterial Community

Functional prediction based on PICRUSt2 identified the 20 most abundant KEGG Orthologs (KOs), which were classified into nine KEGG Level 2 functional categories (Supplementary Figure S4). Hierarchical clustering differentiated the mineral spring samples according to similarities in their predicted functional profiles, revealing variation in the relative abundance of individual KOs among sampling sites. The predicted KOs were assigned to Transporters, Transcription machinery, Membrane transport, Signal transduction, Carbohydrate metabolism, Lipid metabolism, Protein kinases, Poorly characterized functions, and Unclassified metabolism. Among these categories, Transporters included the largest number of predicted KOs, followed by Transcription machinery, Membrane transport, Signal transduction, Lipid metabolism, and Carbohydrate metabolism, whereas Protein kinases, Poorly characterized functions, and Unclassified metabolism contained fewer predicted KOs (Supplementary Figure S4).

**Figure 6:**
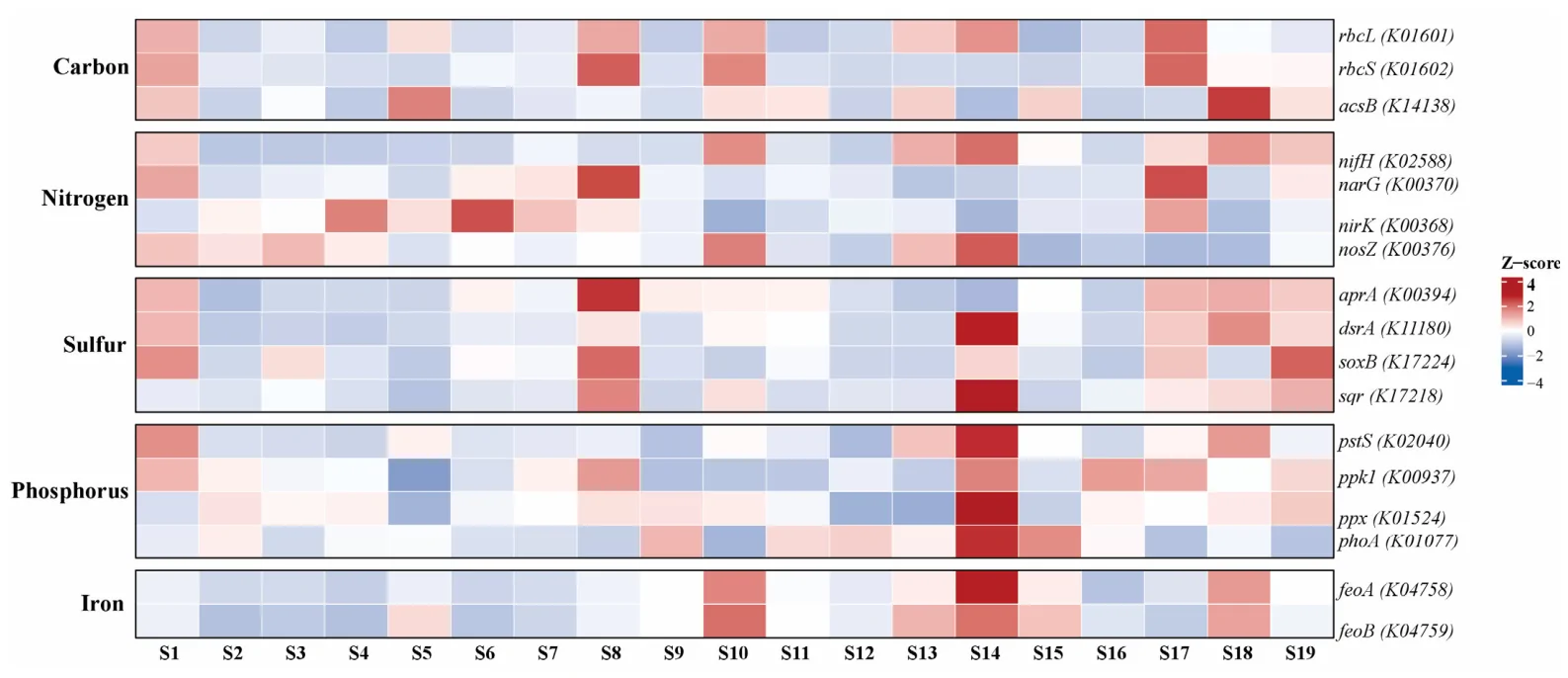
Predicted functional potential associated with selected biogeochemical processes across the 19 mineral spring bacterial communities.

To further examine predicted functions of biogeochemical relevance, 17 KOs were selected from the complete PICRUSt2-predicted functional profile and grouped according to their association with carbon, nitrogen, sulfur, and phosphorus transformations, as well as ferrous iron uptake (Figure 6). Carbon-related predictions included *rbcL* (K01601) and *rbcS* (K01602), associated with CO_2_ fixation through the Calvin cycle, and *acsB* (K14138), associated with the reductive acetyl-CoA (Wood–Ljungdahl) pathway. Nitrogen-related predictions included *nifH* (K02588), associated with nitrogen fixation; K00370 (*narG/narZ/nxrA*), associated with nitrate reductase/nitrite oxidoreductase functions; *nirK* (K00368), associated with nitrite reduction; and *nosZ* (K00376), associated with nitrous oxide reduction.

Sulfur-related predictions included *aprA* (K00394) and *dsrA* (K11180), associated with dissimilatory sulfate/sulfite reduction, together with *soxB* (K17224) and *sqr* (K17218), associated with sulfur and sulfide oxidation, respectively. Phosphorus-related predictions included *pstS* (K02040), associated with phosphate transport; *ppk1* (K00937) and *ppx* (K01524), associated with polyphosphate metabolism; and *phoA* (K01077), encoding alkaline phosphatase. Iron-related predictions were represented by *feoA* (K04758) and *feoB* (K04759), associated with ferrous iron transport. Collectively, these predictions indicate functional potential related to nutrient acquisition and multiple biogeochemical transformations across the mineral spring bacterial communities. Because these functions were inferred from 16S rRNA gene profiles using PICRUSt2, they represent predicted metabolic potential rather than direct evidence of gene expression or in situ metabolic activity.

## 4. Discussion

### 4.1. Hydrogeochemical Heterogeneity of Mineral Springs

The physicochemical characterization of these mineral springs was previously reported by Leiva-Tafur et al. [39] and provides the hydrogeochemical framework for the present study. Building on this previous characterization, the present study focuses on newly generated microbial community data, including bacterial diversity and taxonomic composition based on 16S rRNA gene sequencing, community–environment relationships, microbial association networks, and PICRUSt2-based predictions of functional potential. Thus, the contribution of this study lies in integrating the previously characterized hydrogeochemical context with microbial community structure and predicted functional attributes across these Andean–Amazonian mineral springs.

The mineral springs evaluated in the Andean–Amazonian transition zone of northern Peru exhibited marked hydrogeochemical heterogeneity, reflected by the broad gradients in temperature, pH, electrical conductivity, total dissolved solids, hardness, sulfate concentration, and trace elements. Rather than being characterized by extreme thermal conditions, these springs displayed considerable variability in their chemical and ionic composition, revealing the coexistence of environments with different degrees of mineralization. This heterogeneity is consistent with the geological and environmental complexity of the region, where tectonic activity, lithological diversity, hydrological dynamics, and climatic gradients influence the composition of groundwater [28,32,33].

The subduction of the Nazca Plate beneath the South American Plate, together with the predominance of carbonate and siliciclastic sedimentary rocks, promotes complex water–rock interactions that regulate the availability of dissolved minerals [29,30]. In particular, carbonate formations contribute to increased hardness and alkalinity, whereas evaporitic and sulfur-rich sequences may explain the elevated sulfate concentrations observed in some mineral springs, generating contrasts in the degree of mineralization and the availability of reduced inorganic compounds [47].

These processes are further modulated by the hydrological dynamics of the Marañón River basin and the pronounced climatic and altitudinal gradients of the region, which influence groundwater residence time, the intensity of water–rock interactions, and the ionic composition of the mineral springs [31,48,49,50,51]. Similar relationships among hydrogeochemistry, altitude, and microbial community structure have been reported in geothermal systems in Chile, Bolivia, and Iceland [20,52].

Compared with high-temperature geothermal systems such as Yellowstone (United States), El Tatio (Chile), Tengchong (China), and Iceland, where temperatures frequently exceed 70–90 °C [2,8,23,24], the mineral springs evaluated in this study exhibited a more moderate thermal regime but pronounced chemical and ionic variability. The observed differences in pH, sulfate, sodium, chloride, electrical conductivity, total dissolved solids, and hardness suggest the presence of multiple hydrogeochemical niches associated with varying degrees of mineralization, which may act as environmental filters for different microbial groups [22,53,54].

### 4.2. Taxonomic Composition Reflects Sulfur-, Nitrogen-, and Carbon-Cycling Communities

The taxonomic composition of the bacterial communities revealed marked spatial heterogeneity among the mineral springs of the Andean–Amazonian transition zone, consistent with the hydrogeochemical variability observed across the study area. The predominance of families associated with the sulfur, nitrogen, and carbon cycles, including Desulfobacteraceae, Desulfosarcinaceae, Chlorobiaceae, Nitrosomonadaceae, Nitrospiraceae, and Rhodobacteraceae, suggests that these processes form the basis of the biogeochemical functioning of these ecosystems. Similar community assemblages have been reported in geothermal and mineralized environments, where hydrogeochemical gradients promote the coexistence of microorganisms with complementary metabolic functions [53,55].

Microorganisms associated with the sulfur cycle constituted one of the dominant groups. The abundance of *Desulfobacteraceae* and *Desulfosarcinaceae*, together with the presence of *Chlorobium*, *Sulfurovum*, and *Thiothrix*, suggests that sulfur reduction and oxidation are important processes in these sediments. The coexistence of microorganisms with complementary metabolic strategies is consistent with the presence of redox microenvironments generated by hydrogeochemical heterogeneity, a pattern widely documented in geothermal systems and sulfur-rich mineral springs [56,57,58,59].

The families *Nitrosomonadaceae* and *Nitrospiraceae* indicate the occurrence of nitrification processes, whereas *Rhodobacteraceae* and the phototrophic genus *Oscillochloris* reflect a high degree of metabolic versatility associated with carbon transformation. The simultaneous presence of these groups suggests that local variations in water chemistry promote the coexistence of multiple metabolic strategies, consistent with observations from other geothermal ecosystems [59,60,61].

The detection of *Halanaerobium* and *Haloarcula* in several mineral springs from Bongará suggests the presence of localized niches with higher degrees of mineralization or osmotic stress. The occurrence of these microorganisms is consistent with the variability observed in the ionic composition of some springs and expands the potential functional diversity of these communities. It also highlights their biotechnological potential as sources of extremozymes, exopolysaccharides, and other metabolites of industrial interest [60,61,62].

### 4.3. Environmental Filtering and Community Differentiation

Microbial diversity in geothermal ecosystems and mineral springs is primarily determined by environmental heterogeneity, where the interaction of hydrogeochemical gradients generates diverse ecological niches and shapes microbial community structure [63,64]. Consistent with this ecological framework, alpha diversity analyses of the mineral springs in the Andean–Amazonian transition zone revealed variability among sites, although no statistically significant differences were detected among the hydrogeochemical categories evaluated. This pattern may reflect greater local environmental heterogeneity and, consequently, a broader availability of ecological niches for bacterial communities. Similar patterns have been reported in geothermal ecosystems from different regions of the world, where microscale heterogeneity in oxygen availability, sulfur compounds, dissolved minerals, and organic substrates promotes the coexistence of metabolically distinct microorganisms, maintaining high bacterial diversity even under contrasting hydrogeochemical conditions [41,65].

Although alpha diversity remained relatively consistent across the hydrogeochemical categories, beta diversity analyses revealed differences in bacterial community composition among the mineral springs. The significant result obtained with the Bray–Curtis metric, together with the absence of significant differences based on Jaccard and UniFrac distances, suggests that hydrogeochemical variation is primarily associated with shifts in the relative abundance of taxa rather than with pronounced taxonomic or phylogenetic turnover. This pattern is consistent with observations from geothermal ecosystems in different regions, where physicochemical gradients influence the relative dominance of microbial taxa while the regional assemblage remains largely conserved [52].

Environmental filtering is widely recognized as one of the principal ecological processes governing the assembly of microbial communities in geothermal ecosystems, where multiple physicochemical factors simultaneously constrain the establishment and persistence of microbial populations [65,66,67]. Consistent with this ecological framework, the significant global RDA model indicates a detectable multivariate association between bacterial community composition and the combined hydrogeochemical gradient. However, the relatively low proportion of total community variation accounted for by the constrained model (R^2^ = 0.120; adjusted R^2^ = 0.010) indicates that the measured hydrogeochemical variables explain only a limited component of the overall variation in community composition. This suggests that additional unmeasured environmental factors, local habitat characteristics, stochastic processes, or biotic interactions may also contribute to structuring these bacterial communities [64,65].

### 4.4. Microbial Interactions and Predicted Functional Potential

In addition to environmental filtering, patterns of microbial co-occurrence can provide further insight into the organization of bacterial communities. In the mineral springs studied here, the network showed a structured pattern of associations among ASVs, with a small number of highly connected taxa occupying central positions. ASVs affiliated with *Halanaerobium*, *Anaerophaga*, *Desulfotignum*, and other members of Halanaerobiaeota, Desulfobacterota, and Bacteroidota were among the most highly connected taxa, suggesting that these groups may represent potential hubs within these microbial communities. The three modules identified by the Louvain algorithm further indicate that different groups of ASVs tend to co-occur across the sampled springs. However, these patterns should be interpreted with caution because positive correlations do not necessarily represent direct ecological interactions or mutualistic relationships [68,69]. They may also reflect shared environmental preferences, similar ecological niches, or responses to environmental factors not measured in this study. Thus, the network highlights consistent co-occurrence patterns and taxa of potential ecological relevance, while their functional roles and direct interactions require further experimental validation [22,64,70,71,72].

The targeted functional predictions provide additional evidence of the metabolic potential of these bacterial communities in relation to major biogeochemical processes. Carbon-related KO_s_ included *rbcL* and *rbcS*, associated with CO_2_ fixation through the Calvin cycle, together with *acsB*, associated with the reductive acetyl-CoA (Wood–Ljungdahl) pathway. These predictions suggest the potential occurrence of alternative mechanisms of inorganic carbon assimilation within the mineral spring communities. This functional potential is consistent with the occurrence of bacterial lineages containing phototrophic or chemolithotrophic representatives, including *Chlorobium*, *Oscillochloris*, and *Sulfurovum*. Similar links between microbial community composition, carbon-related processes, and environmental conditions have been described in geothermal systems [65,73].

Predicted nitrogen-related functions included *nifH*, associated with nitrogen fixation; K00370 (*narG/narZ/nxrA*), associated with nitrate reductase/nitrite oxidoreductase functions; *nirK*, associated with nitrite reduction; and *nosZ*, associated with nitrous oxide reduction. Sulfur-related predictions included *aprA* and *dsrA*, associated with dissimilatory sulfate/sulfite reduction, together with *soxB* and *sqr*, associated with sulfur and sulfide oxidation. The coexistence of predicted functions associated with different biogeochemical transformations is consistent with the metabolic diversity described in geothermal microbial communities, where interconnected elemental transformations may contribute to ecosystem functioning [74,75].

Phosphorus-related KOs, including *pstS*, *ppk1*, *ppx*, and *phoA*, indicate predicted capacities associated with phosphate acquisition, polyphosphate metabolism, and phosphatase activity. In contrast, *feoA* and *feoB* are associated with ferrous iron transport and therefore indicate potential for iron acquisition rather than direct evidence of microbial iron oxidation or reduction. Overall, the correspondence between taxonomic composition and predicted functional profiles suggests that these bacterial communities harbor functional potential relevant to carbon, nitrogen, sulfur, and phosphorus transformations and iron acquisition. However, because these functions were inferred from 16S rRNA gene profiles using PICRUSt2, they should be interpreted as predicted metabolic potential rather than as evidence of gene expression or in situ biogeochemical process rates [76].

### 4.5. Ecological Significance, Biotechnological Implications and Future Perspectives

The integration of taxonomic composition, co-occurrence network analysis, and predicted functional profiles provides a comprehensive ecological perspective on the bacterial communities inhabiting the sediments of mineral springs in the Andean–Amazonian transition zone. The predominance of fermentative, sulfate-reducing, and heterotrophic microorganisms, together with the predicted functional potential associated with genome maintenance, environmental signal perception, energy conservation, and carbon transformation, suggests that these communities may play an important role in the biogeochemical processes of mineral-rich environments [52,72]. Furthermore, the presence of diverse bacterial lineages adapted to heterogeneous hydrogeochemical conditions highlights these mineral springs as potential reservoirs of microbial diversity and metabolic capabilities that may represent valuable resources for future research in environmental biotechnology and the exploration of enzymes and metabolic pathways adapted to extreme conditions [25,26,27,75].

An important limitation of the present study is that redox potential (ORP/Eh) and organic and inorganic carbon concentrations were not measured during the original sampling campaign and therefore could not be incorporated into the environmental analyses. Although dissolved oxygen was measured in the spring water, it should not be considered a direct substitute for ORP/Eh. Consequently, the influence of redox conditions and carbon availability on bacterial community structure and associated biogeochemical processes cannot be fully resolved from the present dataset. Future studies should incorporate direct measurements of ORP/Eh, total organic carbon (TOC), dissolved organic carbon (DOC), and dissolved inorganic carbon (DIC) to provide a more comprehensive assessment of the environmental controls on microbial community structure and function in these mineral springs. Another limitation was the absence of independent biological replicates within each spring, which prevents the assessment of small-scale microbial variability. Future studies should incorporate independent replicates at each site to strengthen the assessment of this variability and comparisons among environments.

Nevertheless, because the functional profiles were inferred from 16S rRNA gene sequences using PICRUSt2, future studies integrating shotgun metagenomics, metatranscriptomics, culture-based approaches, and physiological characterization will be necessary to validate the predicted functions and to provide a deeper understanding of the ecological interactions and metabolic capabilities of the microbial communities inhabiting these still poorly explored ecosystems [74,76]. In this context, the present study expands current knowledge of the bacterial diversity and metabolic potential of mineral springs in the Andean–Amazonian transition zone and provides an ecological baseline for future research aimed at understanding the role of these microorganisms in biogeochemical cycles and evaluating their potential as sources of novel microbial resources for environmental and biotechnological applications.

## 5. Conclusions

The mineral springs of the Andes–Amazon transition harbor diverse and spatially heterogeneous bacterial communities associated with the hydrogeochemical variability of these ecosystems. Although alpha diversity did not show significant differences among the evaluated categories, bacterial composition did show differences among thermal categories, particularly between cold and mesothermal springs as well as between hypothermal and mesothermal springs. The RDA confirmed a significant association between bacterial composition and the hydrogeochemical gradient, although the proportion of explained variation was limited, indicating that other environmental and ecological factors also contribute to the organization of these communities.

Among the main findings was the presence of bacterial lineages related to carbon, nitrogen, and sulfur transformations, together with a structured co-occurrence network in which *Halanaerobium*, *Anaerophaga*, and *Desulfotignum* were among the most connected taxa. Functional analysis using PICRUSt2 complemented these results by revealing metabolic potential associated with carbon fixation, nitrogen and sulfur transformations, phosphorus acquisition, polyphosphate metabolism, and ferrous iron uptake. These findings show that the mineral springs of northern Peru constitute environments of interest for understanding how hydrogeochemical heterogeneity is related to microbial diversity and to functions relevant to biogeochemical cycles.

An important limitation was the absence of direct measurements of ORP/Eh and organic and inorganic carbon, which restricts a more complete interpretation of the influence of redox conditions and carbon availability on bacterial communities. Likewise, one composite sample per spring was analyzed, without biological replicates within each site, and metabolic functions were inferred from 16S rRNA gene data using PICRUSt2; therefore, they represent predicted functional potential and not direct evidence of gene expression or in situ biogeochemical activity.

Despite these limitations, the study provides an ecological basis for understanding bacterial communities that remain poorly studied in the mineral springs of the Andes–Amazon transition. Future incorporation of redox and carbon measurements, greater spatial replication, and metagenomic and experimental approaches will allow validation of the identified functions and further investigation of the role of these microorganisms in biogeochemical cycles, as well as evaluation of their potential for future environmental and biotechnological applications.

## Figures and Tables

**Table 1:** Descriptive statistics of physicochemical, microbiological, and trace element parameters in mineral spring waters.

Parameter	Unit	Minimum	Maximum	Mean ± SD
Temperature	°C	15.1	38.2	24.500 ± 6.984
pH	—	5.2	8.722	6.721 ± 0.791
Dissolved Oxygen (DO)	mg L^−1^	0.265	9.19	3.146 ± 2.596
Electrical Conductivity	µS cm^−1^	0.05	253	55.271 ± 88.714
Total Dissolved Solids (TDS)	mg L^−1^	0.022	162.5	27.051 ± 46.863
Total Solids (TS)	mg L^−1^	0.053	319.164	57.750 ± 101.757
Total Suspended Solids (TSS)	mg L^−1^	0	7.09	0.733 ± 1.660
Hardness	mg CaCO_3_ L^−1^	0.01	222.75	50.299 ± 74.608
Alkalinity	mg CaCO_3_ L^−1^	0.009	122.98	11.755 ± 33.713
Chloride	mg L^−1^	0.034	330	68.568 ± 107.103
Sulfate	mg L^−1^	22.21	184.5	94.607 ± 39.676
Sodium (Na)	mg L^−1^	1.71	393.51	120.290 ± 119.561
Potassium (K)	mg L^−1^	0	3.33	0.506 ± 0.884
Magnesium (Mg)	mg L^−1^	0.07	71.36	8.825 ± 19.191
Calcium (Ca)	mg L^−1^	0.64	85.4	19.546 ± 24.715
Iron (Fe)	mg L^−1^	0	0.83	0.363 ± 0.207
Manganese (Mn)	mg L^−1^	0.01	0.62	0.096 ± 0.148
Copper (Cu)	mg L^−1^	0	5.39	0.556 ± 1.320
Zinc (Zn)	mg L^−1^	0	0.54	0.074 ± 0.130
Lead (Pb)	mg L^−1^	0	0.35	0.043 ± 0.090
Antimony (Sb)	mg L^−1^	0	0.45	0.066 ± 0.114
Strontium (Sr)	mg L^−1^	0	8.82	1.101 ± 2.163
Aluminum (Al)	mg L^−1^	0.11	27.66	2.556 ± 5.967
Arsenic (As)	mg L^−1^	0	4.59	1.312 ± 0.918

## Data Availability

The original contributions presented in this study are included in the article/Supplementary Material. Further inquiries can be directed to the corresponding authors.

## Supplementary Materials

The following supporting information can be downloaded at: https://www.mdpi.com/article/10.3390/microorganisms14092106/s1. Supplementary Figure S1: Relative abundance of dominant bacterial genera across the six provinces containing the sampled mineral springs in northern Peru; Supplementary Figure S2: Comparison of alpha diversity indices among temperature, pH, and mineralization classes; Supplementary Figure S3: Principal coordinate analysis (PCoA) based on Bray–Curtis dissimilarity of bacterial communities according to temperature, pH, hardness, and mineralization classes; Supplementary Figure S4: Predicted functional profile of bacterial communities across the 19 mineral spring samples; Supplementary Table S1: Geographic location and elevation of the 19 mineral spring sampling sites in northern Peru; Supplementary Table S2: Alpha diversity indices of bacterial communities across mineral spring sampling sites; Supplementary Table S3: Kruskal–Wallis test results for alpha diversity indices of bacterial communities across physicochemical categories of mineral springs; Supplementary Table S4: Pairwise PERMANOVA results for bacterial community composition based on Bray–Curtis, Jaccard, and UniFrac distance matrices across physicochemical categories; Supplementary Table S5: PCA loadings of the 18 environmental variables retained for the environmental ordination used in the redundancy analysis (RDA).
