# Supplementary material for: Diversity and Structural Composition of Bacterial Communities in Mineral Springs of the Andean–Amazonian Transition Zone in Northern Peru
Source: Microorganisms. 2026 Sep 20;14(9):2106. doi: 10.3390/microorganisms14092106 (PMC13609451; doi:10.3390/microorganisms14092106)
Supplement: Supplementary file 1 [file microorganisms-14-02106-s001.zip › microorganisms-4405152-supplementary.pdf]

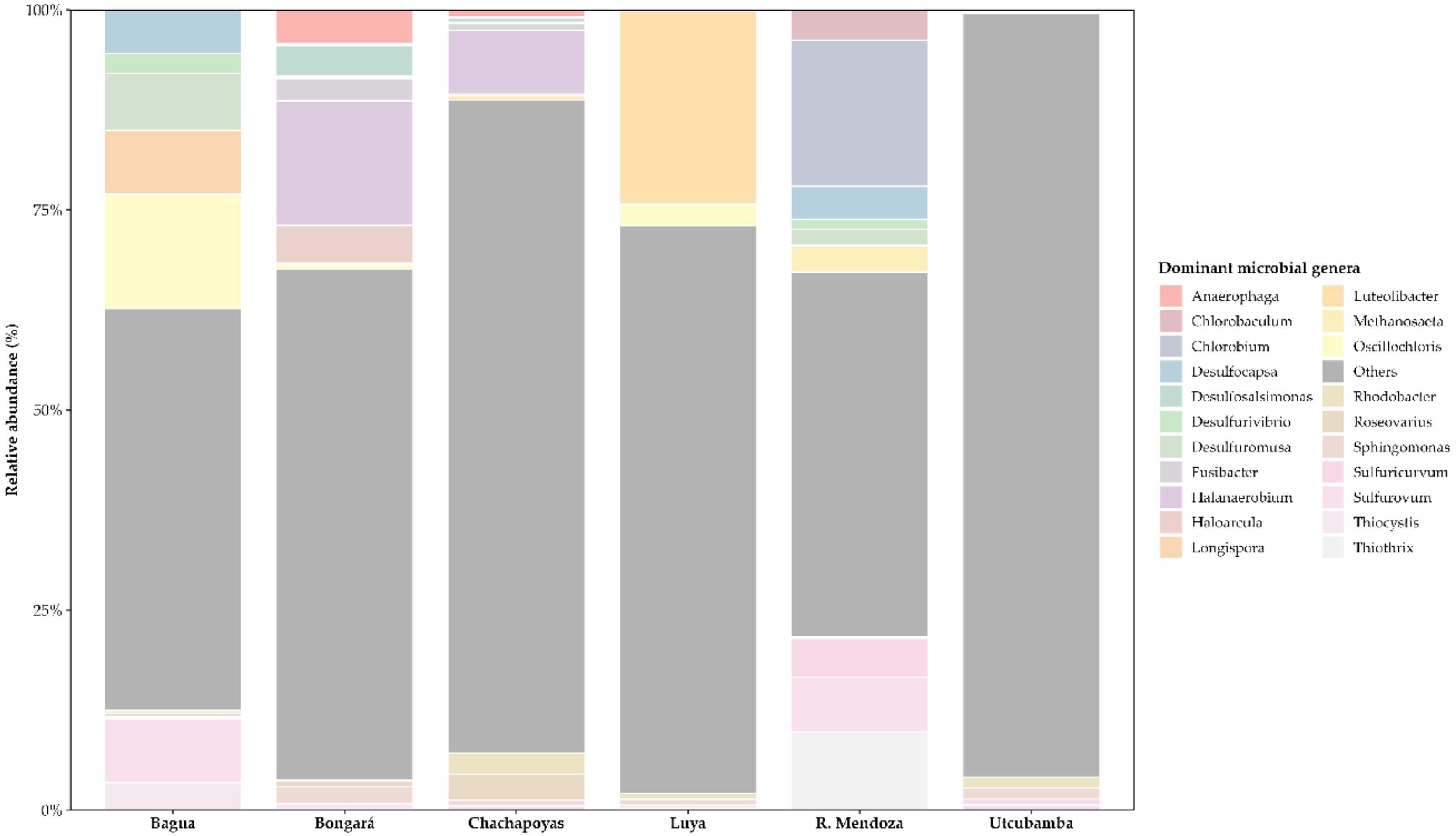

Supplementary Figure S1. Relative abundance of dominant bacterial genera across the six provinces containing the sampled mineral springs in northern Peru. Stacked bars show the relative abundance (%) of dominant bacterial genera in Bagua, Bongará, Chachapoyas, Luya, Rodríguez de Mendoza, and Utcubamba. Low-abundance genera not individually represented are grouped as “Others”.

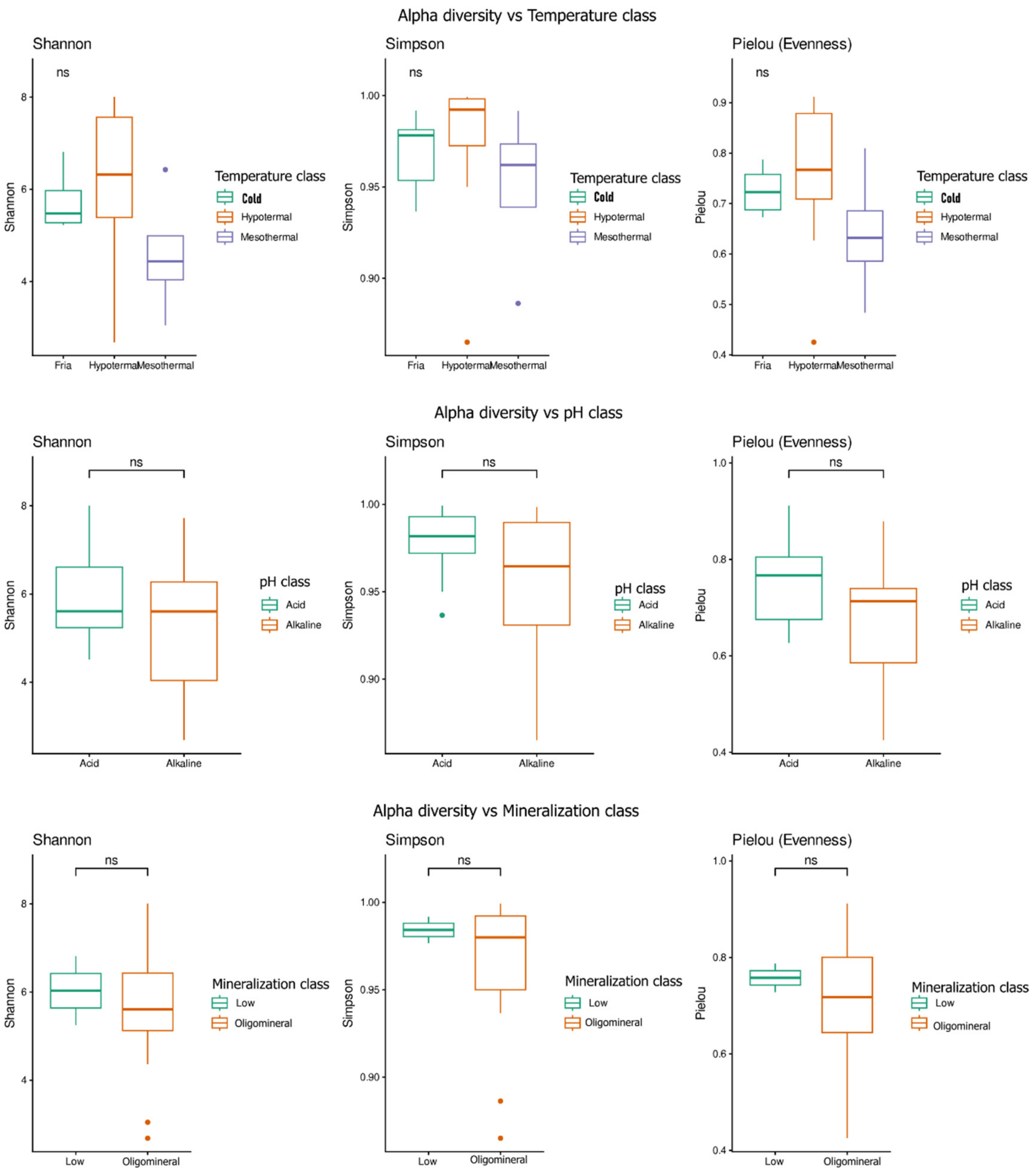

Supplementary Figure S2. Comparison of alpha diversity indices among temperature, pH, and mineralization classes.

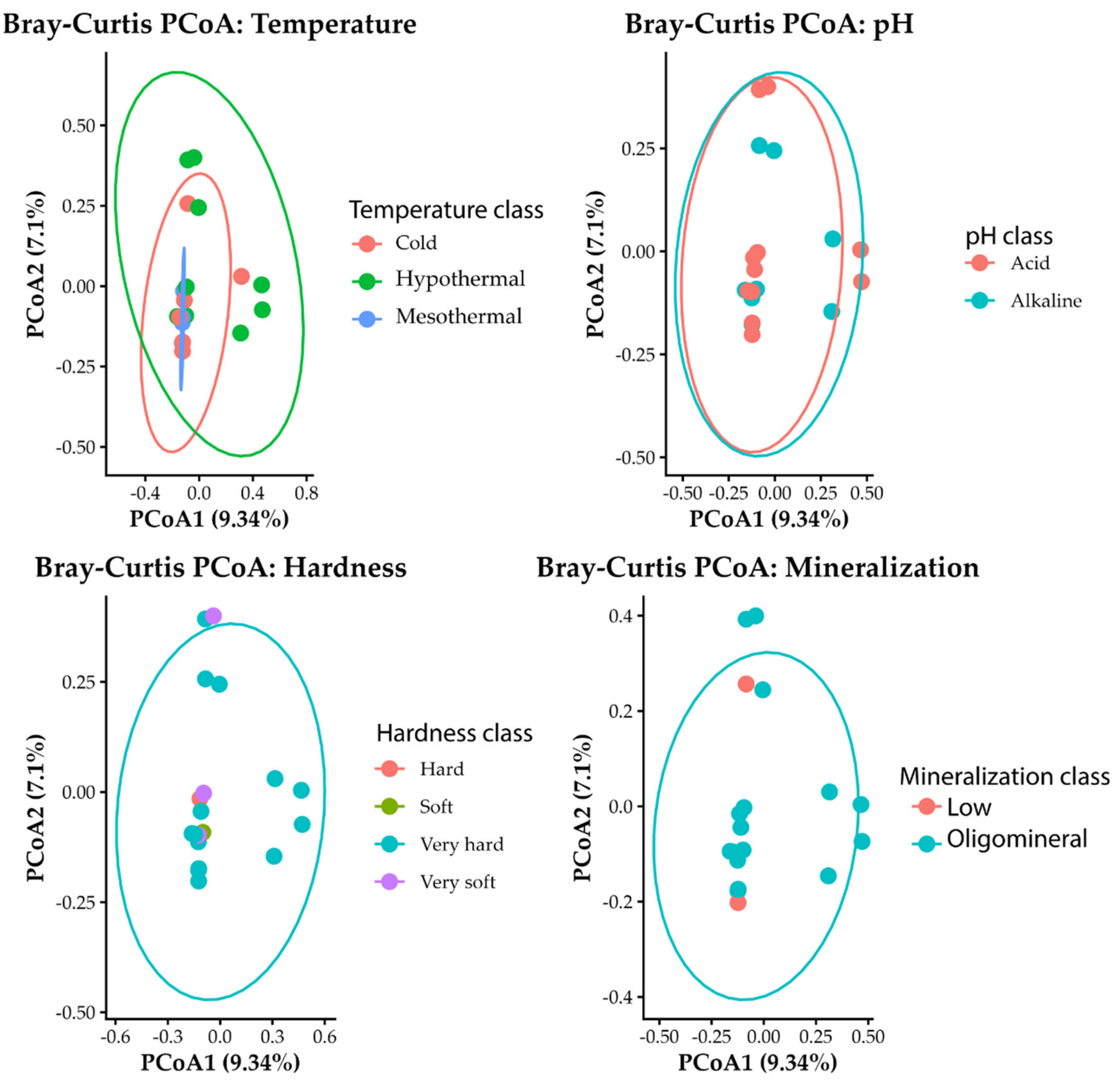

Supplementary Figure S3. Principal coordinate analysis (PCoA) based on Bray–Curtis dissimilarity of bacterial communities according to temperature, pH, hardness, and mineralization classes.

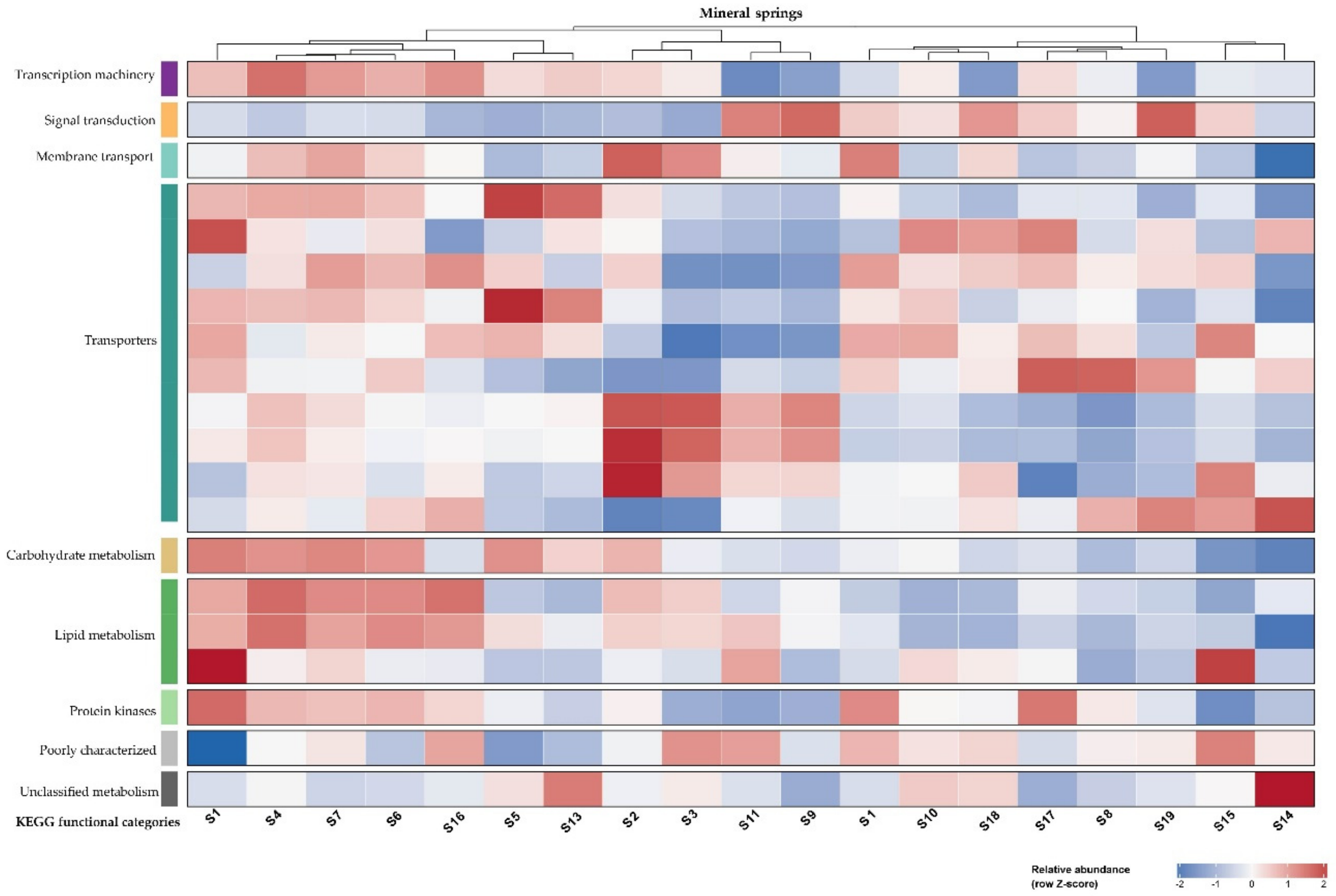

Supplementary Figure S4. Predicted functional profile of bacterial communities across the 19 mineral spring samples. Heatmap showing the relative abundance (row Z-score) of the 20 most abundant PICRUSt2-predicted KEGG Orthologs (KOs), grouped according to KEGG Level 2 functional categories. Hierarchical clustering of samples reflects similarities in their predicted functional profiles.

Supplementary Table S1. Geographic location and elevation of the 19 mineral spring sampling sites in northern Peru.

Sampling site	Province	Spring name	UTM Easting (m)	UTM Northing (m)	UTM Zone	Elevation (m a.s.l.)
S1	Bagua	Aramango	779849	9398066	17S	384
S2	Bagua	Rentema 1	772230	9393075	17S	518
S3	Bagua	Rentema 2	772410	9392931	17S	644
S4	Bongará	Chaquil	171449	9346559	18S	1495
S5	Bongará	Corontachaca	831007	9344874	17S	1275
S6	Chachapoyas	Yurmarca 1	189617	9332737	18S	2592
S7	Chachapoyas	Vituya	193478	9326424	18S	2125
S8	Chachapoyas	Cuelcacha	204088	9325688	18S	3147
S9	Chachapoyas	Clich	198092	9327532	18S	2659
S10	Luya	Caclic	178221	9315303	18S	1618
S11	Rodríguez de Mendoza	Michina 1	221042	9294500	18S	1584
S12	Rodríguez de Mendoza	Michina 2	221043	9294768	18S	1590
S13	Rodríguez de Mendoza	Mono 1	260339	9275348	18S	1552
S14	Rodríguez de Mendoza	Mono 2	261722	9275043	18S	1616
S15	Rodríguez de Mendoza	Tocuya	238570	9284039	18S	1376
S16	Utcubamba	Jamalca	799364	9344060	17S	1101
S17	Chachapoyas	Colpar	205873	9312809	18S	2410
S18	Chachapoyas	Molinopampa	205835	9312863	18S	2404
S19	Chachapoyas	Ventilla	215452	9315684	18S	2585

Note: Coordinates are expressed in the WGS84 / UTM coordinate reference system. Sampling sites are located in UTM Zones 17S and 18S. Elevation is expressed in meters above sea level (m a.s.l.).

Supplementary Table S2. Alpha diversity indices of bacterial communities across mineral spring sampling sites.

Sampling site	Diversity indices			
	Pielou evenness	Shannon entropy	Simpson index	Faith's PD
**S1**	0.841	9.061	0.992	194.391
**S2**	0.670	6.203	0.957	77.332

**S3**	0.545	4.368	0.886	47.100
**S4**	0.747	7.710	0.973	134.882
**S5**	0.480	3.851	0.865	49.196
**S6**	0.723	7.570	0.937	107.078
**S7**	0.832	9.505	0.992	217.304
**S8**	0.756	7.464	0.977	103.387
**S9**	0.791	8.033	0.982	108.638
**S10**	0.916	10.724	0.998	229.407
**S11**	0.917	10.555	0.998	181.603
**S12**	0.839	9.462	0.992	182.224
**S13**	0.819	8.843	0.994	161.206
**S14**	0.759	8.529	0.946	171.299
**S15**	0.776	8.193	0.990	130.017
**S16**	0.694	6.418	0.968	91.349
**S17**	0.943	11.122	0.999	199.403
**S18**	0.674	7.167	0.950	159.762
**S19**	0.732	7.397	0.980	132.201

Supplementary Table S3. Kruskal–Wallis test results for alpha diversity indices of bacterial communities across physicochemical categories of mineral springs.

Physicochemical parameter	Alpha diversity index	H statistic	p-value	Statistical significance
Temperature	Pielou evenness	1.316	0.518	ns
	Shannon entropy	2.216	0.330	ns
	Simpson index	1.319	0.517	ns
	Faith's PD	1.817	0.403	ns

pH	Pielou evenness	0.334	0.563	ns
	Shannon entropy	0.027	0.869	ns
	Simpson index	0.170	0.680	ns
	Faith's PD	0.171	0.680	ns
Hardness	Pielou evenness	4.389	0.222	ns
	Shannon entropy	4.614	0.202	ns
	Simpson index	3.932	0.269	ns
	Faith's PD	3.865	0.276	ns
Mineralization	Pielou evenness	0.071	0.790	ns
	Shannon entropy	0.071	0.790	ns
	Simpson index	0.441	0.507	ns
	Faith's PD	0.000	1.000	ns

Supplementary Table S4. Pairwise PERMANOVA results for bacterial community composition based on Bray–Curtis, Jaccard, and UniFrac distance matrices across physicochemical categories.

Variable	Comparison	Metric	pseudo-F	q-value	Significance
**Temperature**	Cold vs Hypothermal	Bray–Curtis	0.994	0.421	ns
	Cold vs Mesothermal	Bray–Curtis	1.134	0.045	*
	Hypothermal vs Mesothermal	Bray–Curtis	1.096	0.045	*

	Cold vs Hypothermal	Jaccard	1.011	0.301	ns
	Cold vs Mesothermal	Jaccard	1.079	0.075	ns
	Hypothermal vs Mesothermal	Jaccard	1.073	0.071	ns
	Cold vs Hypothermal	UniFrac	0.955	0.538	ns
	Cold vs Mesothermal	UniFrac	1.187	0.363	ns
	Hypothermal vs Mesothermal	UniFrac	1.066	0.390	ns
**pH**	Acid vs Alkaline	Bray–Curtis	0.978	0.520	ns
	Acid vs Alkaline	Jaccard	0.977	0.707	ns
	Acid vs Alkaline	UniFrac	1.042	0.335	ns
**Mineralization**	Low mineralization vs oligomineral	Bray–Curtis	0.989	0.590	ns
	Low mineralization vs oligomineral	Jaccard	0.997	0.521	ns
	Low mineralization vs oligomineral	UniFrac	0.895	0.805	ns

Note: q-values correspond to FDR-adjusted p-values obtained from pairwise PERMANOVA comparisons.

* Significant differences ($q < 0.05$).

ns: not significant ($q > 0.05$).

Supplementary Table S5. PCA loadings of the 18 environmental variables retained for the environmental ordination used in the redundancy analysis (RDA).

Environmental variable	PC1	PC2
pH	0.100	0.660
Electrical conductivity	−0.928	0.186
Dissolved oxygen	−0.420	0.303
Temperature	0.828	0.051
Total dissolved solids (TDS)	−0.675	0.242
Total suspended solids (TSS)	−0.821	0.132
Total solids	−0.925	0.171
Hardness	−0.697	−0.158
Alkalinity	−0.079	−0.373
Chloride	−0.970	0.104
Sulfate	0.038	0.521
Al	0.148	−0.481
As	0.311	0.220
Ca	0.289	0.477
Cu	−0.184	−0.248
Fe	0.142	−0.177
K	0.198	0.742
Mg	0.354	0.734

Note: Environmental variables were standardized prior to PCA. PC1 and PC2 explained 30.96% and 15.59% of the environmental variance, respectively (46.54% cumulatively), and were subsequently used as explanatory variables in the RDA. The sign of each loading indicates the direction of association with the corresponding principal component.
